# Irc3 is a mitochondrial DNA branch migration enzyme

**DOI:** 10.1038/srep26414

**Published:** 2016-05-19

**Authors:** Ilja Gaidutšik, Tiina Sedman, Sirelin Sillamaa, Juhan Sedman

**Affiliations:** 1 Institute of Molecular and Cell Biology, University of Tartu, Riia 23b, Tartu 51010, Estonia

## Abstract

Integrity of mitochondrial DNA (mtDNA) is essential for cellular energy metabolism. In the budding yeast *Saccharomyces cerevisiae*, a large number of nuclear genes influence the stability of mitochondrial genome; however, most corresponding gene products act indirectly and the actual molecular mechanisms of mtDNA inheritance remain poorly characterized. Recently, we found that a Superfamily II helicase Irc3 is required for the maintenance of mitochondrial genome integrity. Here we show that Irc3 is a mitochondrial DNA branch migration enzyme. Irc3 modulates mtDNA metabolic intermediates by preferential binding and unwinding Holliday junctions and replication fork structures. Furthermore, we demonstrate that the loss of Irc3 can be complemented with mitochondrially targeted RecG of *Escherichia coli*. We suggest that Irc3 could support the stability of mtDNA by stimulating fork regression and branch migration or by inhibiting the formation of irregular branched molecules.

The mitochondrial genome encodes 8–13 peptides, essential subunits of the respiratory chain and components of the mitochondrial protein synthesis machinery^1^,^2^. Integrity of mitochondrial DNA (mtDNA) is therefore essential for the respiratory growth of eukaryotic cells. In *Saccharomyces cerevisiae*, a large number of nuclear genes are known to influence the maintenance of mitochondrial genome stability; however, most corresponding genes act indirectly by controlling diverse metabolic pathways, mitochondrial morphology, iron homeostasis or mitochondrial gene expression, for example^3^. Meanwhile, the protein factors directly involved in mtDNA metabolism remain poorly characterized. Genetic recombination is an important feature of yeast mtDNA metabolism, well-known for *S. cerevisiae* for several decades^4^,^5^. Following mating of two haploid yeast strains, parental mitochondrial DNA (mtDNA) is rapidly mixed and randomly transmitted to the progeny cells^6^. During zygote formation, extensive recombination occurs between the parental mtDNAs, as indicated by the high frequency of recombinants (20 to 25%) between unlinked mitochondrial markers^6^. Active recombination is reflected in complex physical topology of yeast mtDNA, characterized by high levels of four- and three-way junctions readily detectable by 2D electrophoresis or by electron microscopy^7^,^8^,^9^,^10^,^11^. Homologous recombination could be important for the initiation of mitochondrial DNA replication using either strand invasion or single-strand annealing mechanism^8^,^10^,^12^,^13^. Some experimental evidence suggests that recombination is involved in double-strand break repair in yeast mitochondria^14^,^15^. On contrary, recombination between short direct repeats is also assumed to be responsible for the frequent generation of respiratory deficient *rho*− mutants that stably transmit mitochondrial genomes containing large deletions^16^. It is likely that a similar recombination-based process generates mtDNA deletions that are frequently detected in neuronal cells, heart and skeletal muscle of normal aged people and in Parkinson’s disease patients^17^,^18^.

While recombination appears to play an important role in yeast mtDNA metabolism, little is known about the protein factors including DNA helicases involved in the formation and processing of branched recombination intermediates. Two DNA helicases of the Superfamily I - Pif1 and Hmi1 - have been identified in yeast mitochondria^19^,^20^ and both enzymes have been suggested to play a role in recombination. PIF1 was identified as a nuclear gene affecting recombination between the *wt* and specific *rho*− mitochondrial genomes^21^. Hmi1p has been shown to preferentially unwind DNA substrates containing flap structures that resemble recombination intermediates^22^. However, mechanistic models explaining the roles of Pif1 or Hmi1 in mitochondrial recombination have not been further elaborated.

Surprisingly, branch migration helicases that are important for a variety of recombination-related processes, including recombinational DNA repair, recovery of stalled replication forks and suppression of ectopic recombination, have not been described in yeast mitochondria, where the levels of three- and four-way junctions in DNA are particularly high. The yeast genome does not encode closely related homologs of RuvB or RecG- the prokaryotic branch migration helicases- and none of the yeast nuclear branch migration enzymes has been shown to be targeted to mitochondria.

Our recent study revealed that Irc3 - a dsDNA dependent ATPase of the Superfamily II- is essential for yeast mtDNA maintenance^23^. *irc3*Δ mutant yeast cells accumulated double-stranded breaks in mtDNA and lose the wild-type mitochondrial genome in the course of a few generations of growth on glucose-containing media. Here we demonstrate that Irc3 is a mitochondrial branch migration enzyme.

## Results

### Irc3 modulates the formation of branched mtDNA intermediates

**Figure 1:**
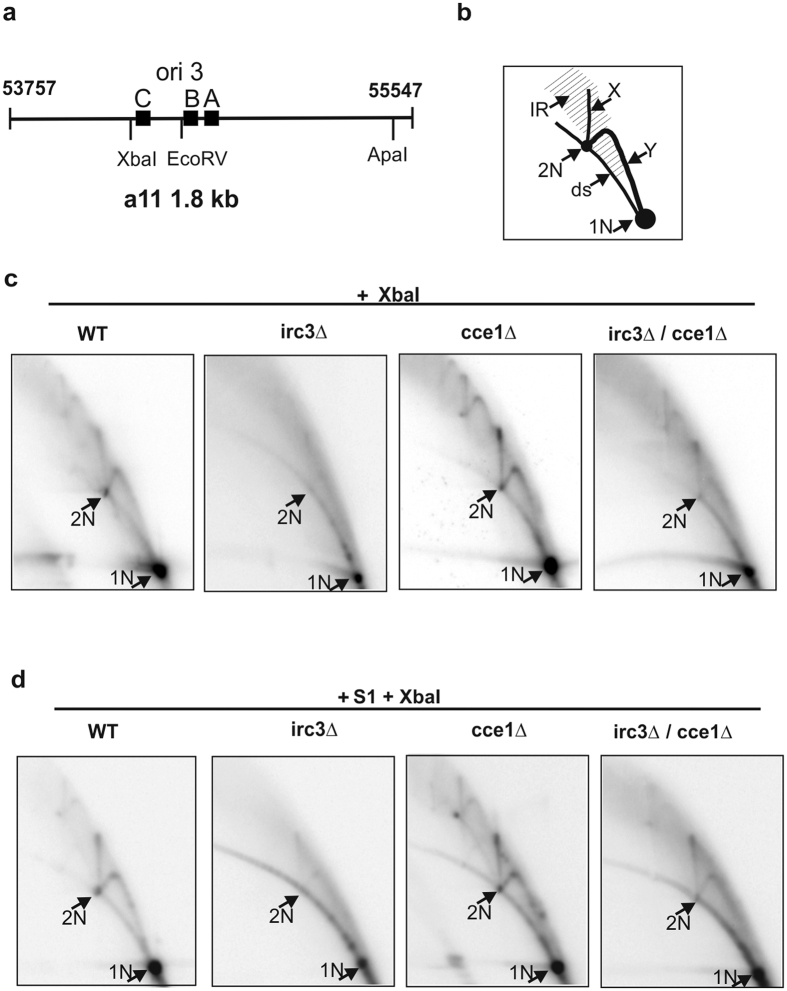
Irc3 modulates the formation of branched intermediates of mitochondrial DNA. (**a**) A map of the 1.8 kb a11 *rho*− mitochondrial DNA (mtDNA) repeat containing actively transcribed *ori3* with characteristic A, B and C boxes. The coordinates correspond to the reference yeast mtDNA sequence (Genbank: KP263414.1). (**b**) A scheme of 2D-gel electrophoresis analysis. ds: linear double-stranded molecules; x: X-arc; y: Y-arc; 1N: unit size (1.8 kb) molecules; 2N: double-unit size (3.6 kb) molecules, IR: irregular branched DNA molecules. (**c,d**) 2D analysis of mtDNA isolated from log phase cultures of the a11 strain with the indicated nuclear mutations. mtDNA was digested with XbaI (**c**) or with S1 and XbaI (**d**) and separated by 2-D agarose gels followed by blot hybridization to the a11 repeat probe.

We have previously reported that Irc3 is directly involved in mtDNA metabolism in both *rho*+ and *rho*− yeast strains^23^. Irc3 is a member of the Superfamily II of helicases and ATP-dependent DNA translocases and *irc3*Δ yeast strains are characterized by excessive fragmentation of mtDNA. This indicates that Irc3 suppresses the formation of dsDNA breaks or is involved in dsDNA break repair, possibly through a recombination-based mechanisms frequently used for dsDNA break repair or by reactivation of stalled replication forks that otherwise can generate dsDNA breaks. Furthermore, our *in silico* analysis revealed that the T4 bacteriophage UvsW helicase is the closest homolog of the *S. cerevisiae* Irc3 protein outside the immediate family of yeast Irc3 orthologs in different yeasts (Supplementary Fig. 1). UvsW is a multifunctional late replication helicase of T4 that drives fork regression and Holliday junction migration^24^,^25^,^26^, suggesting that Irc3 might catalyze similar recombination related transformations. This role of Irc3 was further supported by specific changes of mtDNA branched intermediates observed in *rho*− yeast mutants where disruption of the IRC3 gene does not lead to gross rearrangements in cellular metabolism and could indirectly affect DNA metabolism. Here, we analyzed mtDNA in a respiratory defective *rho*− strain a11, containing a transcriptionally active *ori3* element in the 1.8 kb fragment (Fig. 1, Supplementary Fig. 2). mtDNA, purified from logarithmic yeast cultures was digested with the restriction enzyme XbaI and the resulting DNA fragments were resolved on a neutral 2D agarose gel according to their size and shape (Fig. 1b). In general, branched DNA intermediates move slower in the second dimension of 2D gels compared to linear double-stranded molecules (ds, Fig. 1b) of the same mass. A fraction of partially single-stranded branched yeast mtDNA molecules does not resolve into specific arcs or spots on 2D gels indicating that their structure is irregular (IR, Fig. 1b). To reduce the signal from irregular branched molecules, the DNA samples can be treated with limited amounts of the S1 nuclease that preferentially cleaves single-stranded DNA. Distinctive Y and X arcs usually interpreted as replication forks and Holliday junctions, respectively, were readily detected in a11 mtDNA preparations (Y and X, Fig. 1c, wt). Treatment of a11 mtDNA preparations with the S1 nuclease removed a substantial fraction of the IR signal while leaving the Y and X arcs intact (Fig. 1d, wt). In a11 *irc3*Δ strain, the X and Y arcs were barely detectable on the background of a strong signal from the irregular branched molecules (Fig. 1c, irc3Δ). This result was similar to that previously observed with a different *rho*− strain a1184^23^. Furthermore, in the S1 treated a11 *irc3*Δ mtDNA samples, we could also hardly visualize the regular Y and X structures (Fig. 1d, irc3Δ). To understand if the regular X and Y structures are formed in the absence of Irc3 at all, we next analyzed the a11 *cce1*Δ and a11 *cce1*Δ *irc3*Δ strains. Cce1 is the yeast mitochondrial Holliday junction resolvase^27^. The disruption of Cce1 is expected to block resolution of branched mtDNA intermediates and should facilitate the detection of regular X and Y structures in the *irc3* mutant strain. In accordance with an earlier study^7^, the X arc was more prominent in the a11 *cce1*Δ strain than in *wt* a11 (Fig. 1c, *cce1*Δ). mtDNA blots of the a11 *cce1*Δ *irc3*Δ strain revealed the presence of X and Y arc intermediates; however, their levels relative to irregular branched DNA molecules remained significantly lower compared to the *cce1*Δ a11 strain (Fig. 1c, compare *cce1*Δ and *cce1*Δ *irc3*Δ). Treatment of a11 *cce1*Δ *irc3*Δ mtDNA with the S1 nuclease again reduced the IR signal and clearly visualized the regular X and Y arcs. Compared to a11 *irc3*Δ cells grown in YPD, the cells grown in synthetic medium (SCM) displayed stronger signals corresponding to regular X and Y arc (Compare Supplementary Fig. 2 or Fig. 1 and Supplementary Fig. 3). Remarkably however, reintroduction of Irc3 into the a11 *irc3*Δ strain increased the signals of regular X and Y arcs relative to the signals of irregular branched DNA molecules. (Supplementary Fig. 3). Finally we analyzed the changes of mtDNA copy-number in the tested mutant strains (Supplementary Fig. 4). We found that mtDNA levels were severely affected in in the a11 *irc3*Δ and a11 *cce1*Δ *irc3*Δ strains but also to a lesser extent in the a11 *cce1*Δ strain. All experiments revealing Irc3 dependent changes in mtDNA topology and mtDNA copy-number supported the idea that Irc3 is directly involved in yeast mtDNA metabolism. Our data suggest that disruption of Irc3 leads to the accumulation of irregular branched mtDNA intermediates and could inhibit but not completely block the formation of regular X and Y intermediates.

### Irc3 binds to branched DNA molecules

**Figure 2:**
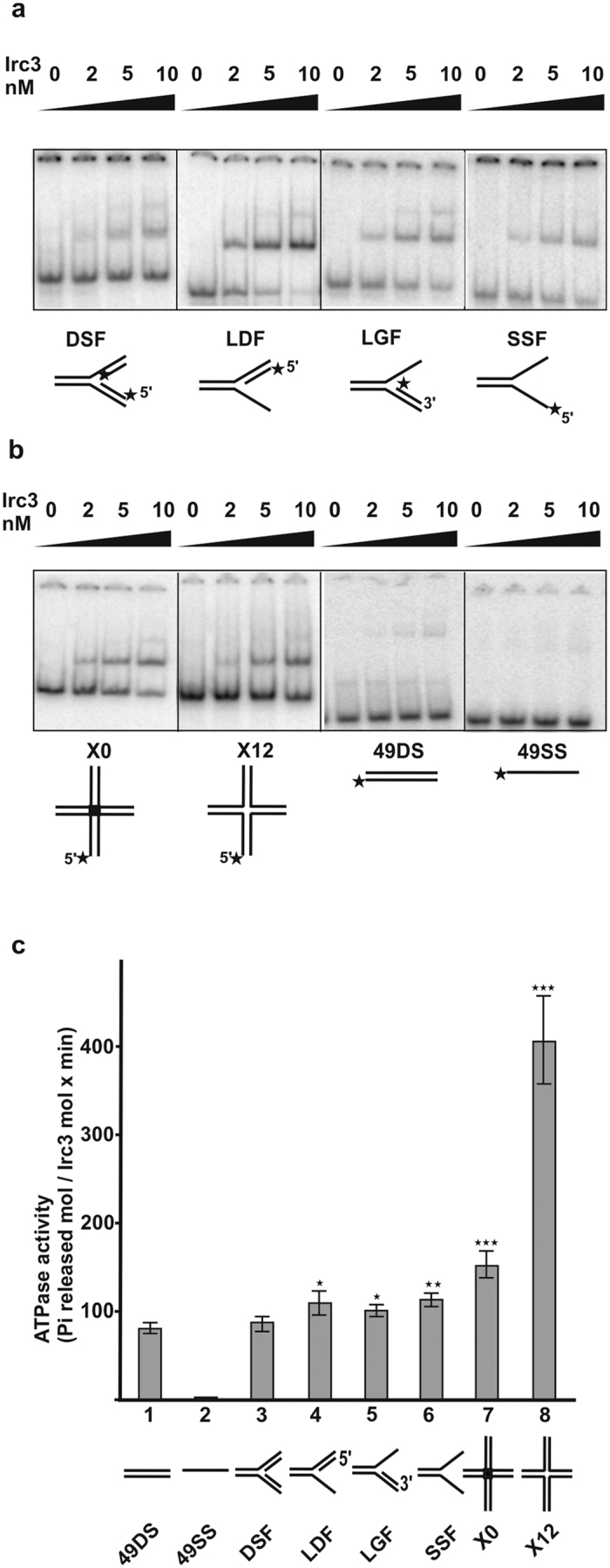
Irc3 binds preferentially to branched-DNA. (**a,b**) Electrophoresis mobility shift assays (EMSA) using indicated concentrations of Irc3 and ^32^P-labeled probes mimicking the structure of a replication fork (**a**) or X-shaped and linear DNA (**b**), as schematically depicted below the panels. Position of the 5′ label in the DNA substrates is indicated by a star. DSF: fork with both arms double-stranded; LDF- fork with missing nascent lagging strand; LGF- fork with missing nascent leading fork, SSF:fork with both arms single-stranded and X0: immovable four-way junction; X12- four-way junction containing a movable core flanked by heterologous sequences of 19 or 20 bp in each arm; 49DS: 49 bp dsDNA; 49SS: 49 nt ssDNA. (**c**) Stimulation of Irc3 ATPase activity with different DNA cofactors described for panels **a**,**b**. All assays were performed as triplicates using 15 nM Irc3 and the error bars indicate SD. The significance of differences between Irc3 ATPase activities stimulated by the linear 49 bp dsDNA and various branched-DNA cofactors was determined using Student’s t-test for unpaired observations. *P < 0.05; **P < 0.01; ***P < 0.001.

Irc3-dependent changes in mtDNA recombination intermediates prompted us to test if Irc3 binds to branched DNA molecules. Therefore, we developed an EMSA assay where Irc3 complex formation with different radiolabelled substrates (Supplementary Table 2) was analyzed in the presence of nonspecific competitor dsDNA, as described in Materials and Methods. We found that in the absence of ATP, Irc3 formed complexes with different branched model substrates, with apparent Kd in the low nanomolar range (Fig. 2a,b, Supplementary Fig. 5). Complex formation was detected with Y-shaped model substrates that mimic a completely double-stranded replication fork and with substrates that had one single-stranded and one double-stranded arm, mimicking structures that would result from incomplete synthesis of the lagging or leading strand, respectively (Fig. 2a). We also detected Irc3 complexes with a Y-shaped DNA substrate that had two single-stranded arms (Fig. 2a). Furthermore, Irc3 formed a complex with two different X-shaped DNA substrates - X0 and X12 - that mimic Holliday junctions (Fig. 2b). X0 represents an immobile structure; however, X12 can undergo limited branch migration, because the central 12 nucleotides are homologous on all branches. Irc3 demonstrated highest affinity towards fork-like structures containing one or two single-stranded arms (Supplementary Fig. 5). Complex formation of short linear dsDNA or ssDNA molecules was marginal under these experimental conditions (Fig. 2b). We concluded that Irc3 preferentially binds to different branched DNA structures and the binding does not require ATP hydrolysis.

We next tested if branched DNA cofactors can stimulate Irc3 ATPase activity (Fig. 2c). Our analysis showed that all tested Y-shaped DNA cofactors appeared to be more effective than previously tested linear dsDNA cofactors of similar length. Moreover, both X-structured cofactors analyzed were found to be even more potent as the ATPase cofactors, especially the X12. As previously reported, the ssDNA cofactor did not stimulate Irc3 ATPase (Fig. 2c and^23^). Preferential binding of Irc3 to Y and X-shaped DNA and strong stimulation of Irc3 ATPase activity with branched DNA cofactors suggested that Irc3 could function as branched DNA specific enzyme.

### Irc3 displays fork reversal and branch migration activity

**Figure 3:**
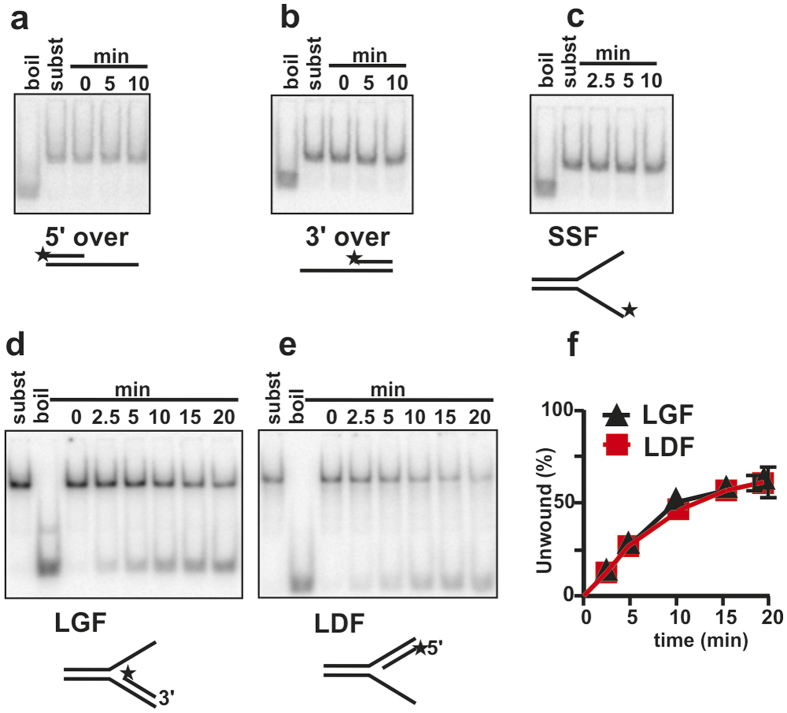
Irc3 unwinds stalled replication forks. (**a,b**) Irc3 is inactive on linear DNA substrate molecules with a 5′ (**a**) and 3′ (**b**) single-stranded extension or with a single-stranded fork structure (**c**). (**d–f**) Irc3 unwinds the lagging- or leading-strand analog from a branched DNA substrates that mimic defective replication forks. LDF- fork missing nascent lagging strand (**d**,**f**); LGF- fork missing nascent leading fork (**e**,**f**). Irc3 was 5 nM in all reactions.

The members of the SFII superfamily can have helicase or nucleic acid translocase activity. In accordance with our previous analysis, Irc3 did not unwind different linear DNA substrates with either 5′ or 3′ single-stranded extensions of 15–25 nt or with a 25 nt single-stranded fork structure at the end of a short double-stranded region (Fig. 3a–c^23^). However, preferential complex formation with branched DNA molecules and strong stimulation of Irc3 ATPase by the same cofactors suggested that Irc3 could unwind substrates resembling four-way Holliday junctions or replication forks. These model substrates were next tested in helicase assays. We first compared fork substrates that had one single-stranded and one double-stranded arm to understand if Irc3 could initiate repair of blocked replication forks (Fig. 3d–f). The substrate LGF (Fig. 3d) mimics a fork where leading strand synthesis is blocked and the single-stranded arm has a free 3′ end. The substrate LDF (Fig. 3e) mimics a fork where the lagging strand synthesis has not been initiated and the single-stranded arm has a free 5′ end. We found that Irc3 can unwind the nascent strand from both substrates (Fig. 3f). Accordingly, Irc3 could assist to initialize fork reversal in different repair situations, when either the leading or lagging strand template is damaged and one or the other nascent strand is missing.

**Figure 4:**
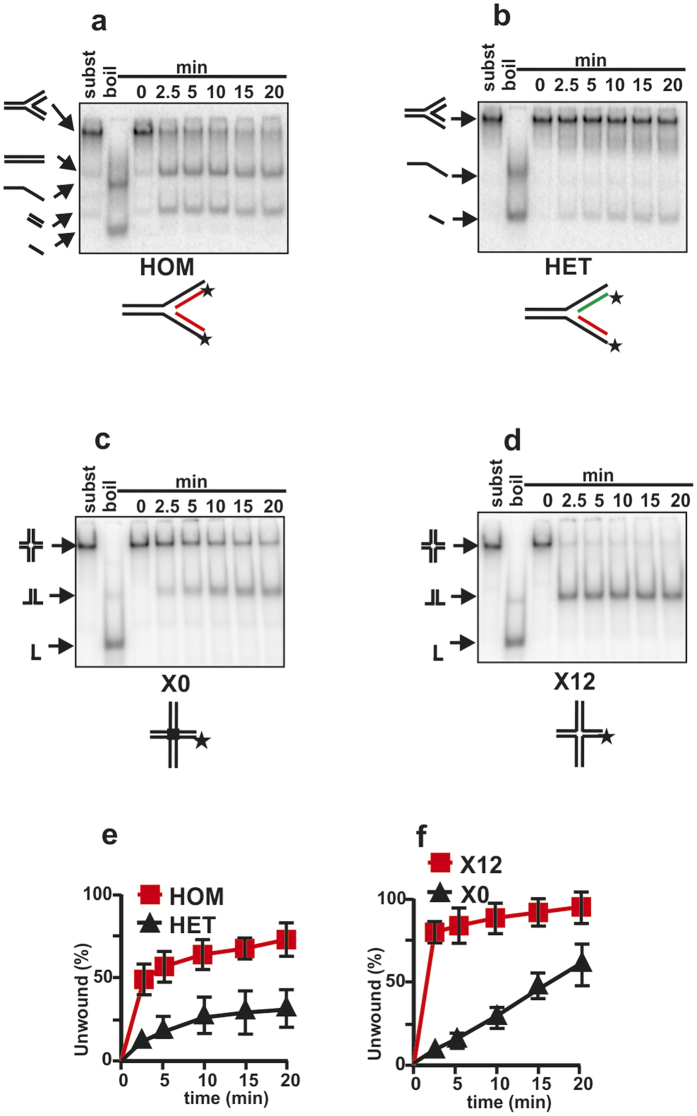
Irc3 preferentially reverses replication fork structures and possesses branch-migrating activity. Comparison of Irc3 dependent unwinding of model fork substrates with homologous (HOM) (**a**,**e**) or heterologous (HET) arms (**b,e**). (**c,d,f**) Irc3 dependent unwinding of four way junctions with fixed immobile structure (**c**,**f**) or junctions with a mobile 12 nt core (**d**,**f**). Irc3 was 5 nM in all reactions.

Next we analyzed branched substrates where all arms were double-stranded (Fig. 4). First we compared two three-way branched replication fork analogs (Fig. 4a,b,e). The arms of the substrate HET are different and the structure can only undergo fork dissolution, whereas the arms of the substrate HOM are homologous and the structure can therefore undergo fork reversal or dissolution. Fork reversal is expected to generate two dsDNA products; fork dissolution releases separated single strands by unwinding the double-stranded arms of the fork. Our experiments demonstrated that Irc3 was active on both substrates (Fig. 4a,b); however, there were significant differences in the rates of reactions (Fig. 4e) as more than 50% of the substrate HOM but less than 10% of the substrate HET was unwound in 2.5 minutes. Unwinding of the HET substrate expectedly produced ssDNA, however the reaction with the HOM substrate resulted in dsDNA product release (Fig. 4b and Supplementary Fig. 6f).

Lastly, we analyzed two model substrates mimicking Holliday junctions - X0 and X12 - in the Irc3-catalyzed unwinding reaction (Fig. 4c,d and f). Both X0 and X12 were unwound by Irc3, the reactions exclusively produced dimeric Y products and no accumulation of single-stranded intermediates was detected (Fig. 4c,d). Importantly, the unwinding reaction of the X12 substrate was much faster compared to X0 (Fig. 4f), as more that 70% of X12 was unwound in 2.5 minutes but only 5% of X0 was unwound by the same time. It is important to note that all Irc3-dependent unwinding reactions are dependent on active ATP hydrolysis as the point-mutation K65A in the Walker A motif of the Irc3 protein completely blocks the unwinding activity (Supplementary Fig. 6). We concluded that Irc3 can catalyze unwinding of branched DNA molecules and the reaction is significantly more effective on substrates that can undergo branch migration.

### RecG partially complements the loss of Irc3

**Figure 5:**
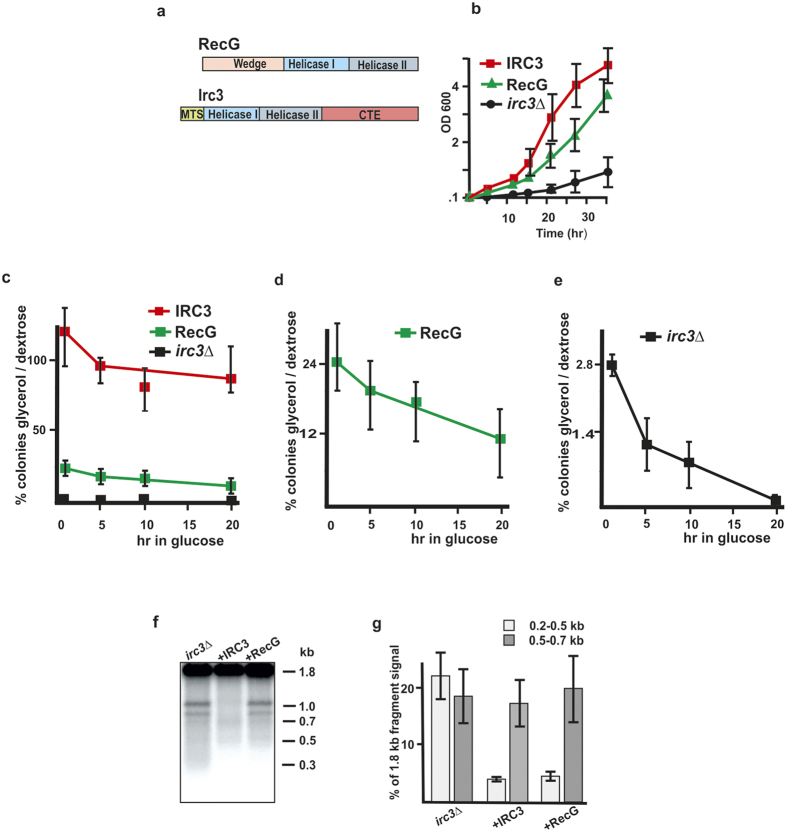
RecG complements partially the loss of Irc3 in yeast mitochondria. (**a**) Graphic representation of RecG and Irc3 sequences. MTS- cleavable mitochondrial targeting sequence; Helicase I and Helicase II- helicase domains; Wedge- RecG wedge domain; CTE- C-terminal extension of Irc3. (**b**) Growth curves of yeast cells in SC –Leu medium containing 3% glycerol as a carbon source. Δ*irc3* + IRC3- red squares; Δ*irc3* + RecG- green triangles; Δ*irc3* + control plasmid- black circles. (**c–e**) Loss of respiratory competence of yeast cells during growth on glucose, as revealed by plating out equal number of cultivated cells onto glycerol and glucose containing agar plates. In d and e, Δ*irc3* + RecG and Δ*irc3* strain viability is shown using different scale of the y axis. Three biological replicas were analyzed and the error bars represent minimum and maximum values of the measurements. (**f**) Mitochondrial DNA fragments in Δirc3 strain of W303-1B background. Mitochondrial DNA was isolated from the indicated strains, digested with EcoRV prior electrophoresis and blot-detection for a 1.8 kb cox2 fragment. Different samples were normalized to equalize the signals of the 1.8 kb fragments. (**g**) Quantification of DNA fragments in the range of 0.2–0.5 kb and 0.5–0.7 kb in the RV digested mtDNA of the indicated strains. Three biological replicas were analyzed and the error bars indicate SD.

The fork reversal and branch migration activities of Irc3 and specific changes in mtDNA branched intermediates in *irc3*Δ yeast cells were consistent with a model that Irc3 is a mitochondrial branch migration enzyme. The biochemical activities of Irc3 described here were similar to that of the double-stranded DNA translocase RecG of *Escherichia coli*, which can catalyze branch migration and replication fork regression to repair stalled replication fork structures containing a leading-strand gap^28^,^29^,^30^. Furthermore, RecG is a conserved protein in prokaryotes and recently found plant homologs of RecG are required for mtDNA stability^31^,^32^,^33^,^34^. While the biochemical activities of Irc3 and RecG appear to be related, the proteins belong to different branches of the large superfamily II and their modular architecture is different. RecG has an additional wedge domain at the N-terminus of the protein and the helicase domain is encoded by the C-terminal part of the molecule^35^. In contrast, Irc3 has an additional domain at the C-terminus (CTE) and the helicase domain is at the N-terminus (Fig. 5a). We decided to test if RecG can complement the deletion of Irc3 by expressing bacterial RecG fused to a Cit1p mitochondrial targeting signal in *irc3*Δ yeast cells. The Cit1-RecG fusion was introduced into yeast cells under the control of CYC1 promoter using the centromeric plasmid pRS315. Our analysis indicated that mitochondrially targeted RecG significantly improved the growth of *irc3*Δ yeast cells on glycerol- containing synthetic media: the doubling times of *irc3*Δ, *irc3*Δ+RecG and *irc3*Δ+IRC3 were approximately 8, 4 and 3 hours, respectively (Fig. 5b). We next compared the stability of the functional *rho*+ mitochondrial genome in the *irc3*Δ, *irc3*Δ+RecG and *irc3*Δ+IRC3 strains by releasing the cells from glycerol containing medium into glucose containing SC-Leu medium where the functional mitochondrial genome is dispensable (Fig. 5c–e). At the indicated times, equal number of cells were plated on glucose and glycerol containing SC – Leu agar plates and viable colonies were scored after 3–5 days of growth. The assay again revealed drastic differences between the *irc3*Δ and *irc3*Δ+IRC3 cells (Fig. 5c,e). Moreover, in the *irc3*Δ+RecG cells, retention of the functional mtDNA was greatly improved compared to *irc3*Δ strain (Fig. 5c–e). However, the stability of mtDNA in the *irc3*Δ+RecG cells still remained devalued compared to the *irc3*Δ+IRC3 strain (Fig. 5c). These tests indicated that RecG can stabilize the functional mitochondrial genome in *irc3*Δ strain. Furthermore, our analysis of mtDNA fragmentation in *irc3*Δ yeast cells by probing a 1.8 kb EcoRV fragment in the cox2 region indicated that the level of 0.2–0.5 kb fragments detected on Southern blots decreases approximately 5-fold when the cells contained a plasmid expressing either Irc3 or RecG (Fig. 5f,g). We conclude that RecG can partially rescue the defects of mtDNA metabolism in *irc3*Δ yeast cells, supporting the idea that Irc3 could be an enzyme involved in yeast mitochondrial replication fork regression or branch migration.

## Discussion

SF II helicases affect diverse aspects of DNA and RNA metabolism. A number of SF II enzymes unwind double-stranded nucleic acid structures, while others translocate on single-stranded or double-stranded nucleic acid without unwinding the substrate. In the yeast *S. cerevisiae*, four members of this large protein family - Irc3, Suv3, Mss116 and Mhr4 - are targeted to mitochondria^23^,^36^,^37^,^38^; however, only Irc3 is a DNA-specific enzyme. *In silico* analysis indicates that Irc3 is conserved in different yeast species, with the closest homolog outside the immediate Irc3 family being the bacteriophage T4 helicase UvsW (Supplementary Fig. 1). Here we demonstrate that Irc3 is a mitochondrial branch-migrating enzyme.

Previously, we found that Irc3 has double-stranded DNA-dependent ATPase activity suggesting that the protein could translocate along double-stranded nucleic acid molecules. The enzymatic properties of Irc3 described here, link the protein specifically with the metabolism of branched mtDNA molecules. Both the yeast and plant mtDNA contain a large number of three- and four-way branches; therefore, helicases remodeling complex DNA structures are expected to be crucial for the integrity of the mitochondrial genome. However, until now the identity of proteins with such enzymatic activity remained unknown in yeast. The plant nuclear genome encodes a homolog of the bacterial RecG helicase that was probably acquired from the chloroplast ancestor and is not present in yeasts^33^,^34^. A number of nuclear SFII enzymes that can catalyze branch migration and fork regression are known in the yeast, including Rad54, Rad5 and RecQ paralogs^39^,^40^,^41^,^42^,^43^. However, none of them have been shown to be targeted to mitochondria. Somewhat surprisingly, the human RECQL4 is found in mitochondria where it affects the integrity of the organellar genome^44^,^45^,^46^. The RECQL4 homolog in yeast- Hrq1p - is a nuclear protein, involved in interstrand crosslinks repair and in addition of telomeric repeats to dsDNA breaks^42^. Here we demonstrate that mitochondrially targeted *E. coli* RecG complements the loss of Irc3 in the yeast. Therefore, Irc3 appears to be the functional homolog of RecG in the yeast mitochondria despite the different domain organization of the two proteins. The presence of a dedicated branch migration helicase in both yeast and plant mitochondria indicates that RecG-type activity is crucial for the metabolism of mtDNA of complex topology.

Our biochemical analysis demonstrates that Irc3 can promote the reversal of model replication forks and the branch migration of Holliday junctions. Interestingly, Irc3 helicase has no unwinding activity on linear partially double-stranded DNA substrates. Additionally, we have shown that ssDNA does not stimulate Irc3 ATPase activity^23^. In contrast, bacterial RecG has been reported to unwind short dsDNA molecules as a helicase of 3′–5′ polarity^47^. Mechanistic differences could thus exist between the DNA remodeling reactions catalyzed by Irc3 and RecG, but nonetheless, Irc3, like RecG, is most likely translocating on double-stranded DNA.

While a number of SFII family enzymes function as dsDNA translocases but are not *bona fide* helicases, Irc3 can act as an unwinding enzyme *in vitro*, but strictly on branched substrates. Therefore, Irc3 differs from the Rad54 protein of the SWI/SNF family that translocate along double-stranded DNA and can only support branch migration but not dissolution of model substrates^48^,^49^,^50^. Irc3 unwinds forked substrates with heterologous branches and X-structures with four different arms, albeit relatively inefficiently. Model fork substrates where either the leading or lagging nascent strand is missing are unwound by Irc3, while RecG has been reported to prefer substrates mimicking stalled leading strand forks^29^,^51^,^52^. It is possible that the substrate specificity of Irc3 in unwinding reactions is modulated by the presence of additional proteins, such as the mitochondrial single-stranded DNA binding protein Rim1, as similar interactions have been observed for the RecG helicase^51^,^53^,^54^.

Our *in vitro* experiments demonstrate that Irc3 can remodel replication forks and Holliday junctions in ATP-dependent manner. These enzymatic characteristics are reminiscent of that of RecG, reviewed in^55^,^56^ and could help to clarify the role of Irc3 in mtDNA maintenance. However, it is notable that in contrast to RecG function in bacterial cells, the Irc3 helicase is essential for yeast wt mtDNA stability as Δ*irc3* mutants lose the *rho*+ genome during few generations of growth on fermentable carbon source^23^. It is therefore possible that Irc3 substitutes the functions of the other bacterial branch-migrating enzyme- RuvB- as well. However, it is also possible that Icr3 activity is essential because mitochondrial DNA is under a constant threat of damaging factors such as ROS produced as a by-product of the respiratory chain reactions. It is thought that RecG is involved in ATP-dependent stalled replication fork regression^29^,^30^,^57^. Lesions in mtDNA can block the mtDNA polymerase, resulting in fork collapse and an accumulation of DNA breaks unless the damage is repaired. Irc3 could play an important role in reactivating replication by fork regression i.e. by helping to move the fork away from the damaged site thus allowing efficient repair.

Our analysis indicates, that in the absence of Irc3, the fraction of irregular branched structures increase in mtDNA. Therefore, in addition to its role in replication fork regression, or alternatively to this, Irc3 appears to inhibit the formation of irregular branched mtDNA molecules, most likely by limiting ectopic recombination. Irc3 could do this by unwinding branched heteroduplex intermediate structures formed on the basis of limited short homology, as also previously suggested for the RecG helicase in bacterial cells and in plant mitochondria^33^,^34^,^58^.

In summary, our data suggest that Irc3 is a branch-migrating enzyme in yeast mitochondria. Disruption of Irc3 leads to the accumulation of complex irregular molecules and DNA breaks associated with eventual loss of the mitochondrial genome. Our studies established that Irc3 is involved in branched mtDNA metabolism. Irc3 appears to be the first yeast DNA helicase which is essential for mtDNA stability maintenance. Hmi1 protein is required for the stability of the *rho*+ genome, however Hmi1 mutants defective in ATP hydrolysis still can support the maintenance of the *rho*+ genome. Therefore the protein probably has an essential structural role in yeast mitochondria^59^. Pif1, the third DNA helicase targeted to yeast mitochondria, is essential for mtDNA maintenance only at elevated temperatures^19^. Interestingly, a hexameric replicative helicase has not been found in yeast mitochondria. Future studies are therefore definitely needed to understand the mechanism of DNA unwinding during mtDNA replication in yeast.

## Methods

### Yeast strains, plasmids and media

The yeast *Saccharomyces cerevisiae* strain a11 is a *rho*− mutant of W303-1B background (Fig. 1a). Δ*irc3* and Δ*cce1* disruption strains of a11 were generated by replacing the ORFs with the HPH or URA3 cassette via homologous recombination.

pRS315-IRC3 plasmid was described in^23^; pRS315-RecG contained the CYC1 promoter (290 nucleotides upstream of ATG), the first 117 nucleotides of *S. cerevisiae* CIT1 ORF as a mitochondrial targeting signal, the full-length *E. coli* RecG ORF and the *S. cerevisiae* CYC1 terminator (272 nucleotides downstream the CYC1 ORF).

Complete medium for yeast growth (YPD) contains 1% Bacto Yeast Extract, 2% Bacto Peptone, 2% glucose. YPG contains 3% glycerol instead of glucose. Strains containing the pRS315 plasmid were propagated in synthetic complete medium -Leu.

### DNA substrates and cofactors

Oligonucleotides used in this study (Supplementary Table 1) were purchased from TAG Copenhagen A/S. The model DNA substrates (Supplementary Table 2) were 5′-end labelled with γ-^32^P ATP (3000 Ci/mmol, Hartmann Analytics GmbH) and polynucleotide kinase. Efficiency of ^32^P- incorporation was measured by spotting an aliquot of the labelling mixture onto DE32 paper and washing with 0.25 M Na-phosphate buffer pH 7.5. Oligonucleotides (0.2 μM) were annealed in 33 mM Tris-Acetate pH 7.9, 66 mM K-Acetate, 10 mM Mg-Acetate. The annealing mixture was heated to 95 °C for 5 min, followed by slow cooling to room temperature. The substrates were purified on non-denaturing 10% PAGE in 89 mM Tris-Borate pH 8.3, 1 mM EDTA (TBE), incubated overnight by shaking in 10 mM Tris-HCl pH 7.5, 1 mM EDTA, 100 mM NaCl (TEN) at 4 °C and precipitated with 4 volumes of ethanol. The purified substrates were stored in TEN at −20 °C. DNA concentrations are indicated in moles of the molecular structure. DNA cofactors for ATPase assays were prepared by annealing equimolar amounts of unlabeled complementary oligonucleotides.

### Purification of Irc3 protein

Irc3 was overproduced in *E. coli* using pGEX4T-1 expression vector (GE Healthcare) as described previously^23^.

### Electrophoresis mobility shift (EMSA) assays

Irc3 and 0.1 nM ^32^P-labelled DNA were mixed in binding buffer (20 mM Tris-HCl pH 7.5, 50 mM NaCl, 1 mM MgCl_2_, 1 mM DTT, 10% glycerol, 1 μg/ml EcoRI digested pUC18 plasmid DNA and 0.5 mg/ml BSA) and incubated on ice for 10 min before loading onto a pre-cooled 5% non-denaturing TBE polyacrylamide gel. Electrophoresis was at 15 V/cm for 1 h at 4 °C. Gels were dried and exposed to phosphoimager Typhoon TL 7500 screens and the data were analyzed with ImageQuant TL software (GE Healthcare).

### DNA unwinding assays

Oligonucleotide-based substrates (0.25 nM) were mixed with Irc3 in helicase assay buffer (20mM Tris-HCl pH 7.0, 1 mM MgCl2, 150 mM NaCl, 1 mM DTT, 1 mM ATP, 0.1 mg/ml BSA, 10% glycerol) and incubated at 30 °C for the indicated times. Unwinding reactions were quenched by the addition of 0.3 volume of 20 mM Tris-HCl pH 8.0, 15 mM EDTA, 0.5% SDS, 0.1% bromophenol blue and 10% glycerol. Reaction products were analyzed on non-denaturing 10% TBE polyacrylamide gels. Electrophoresis was at 10 V/cm for 1.5 h at 4 °C. Gels were dried, exposed to phosphoimager screens and analyzed with ImageQuant TL software.

### ATPase assays

ATPase activity of Irc3 was measured in 5 min reactions and was measured by charcoal binding method as previously described^22^.

### Analysis of mtDNA copy-number, fragmentation and topology

mtDNA copy-number in the hypersuppressive strain a11 was analyzed by probing EcoRV digested total yeast DNA Southern blots as previously described^23^.

The levels of wt mtDNA fragmentation was analyzed by probing EcoRV digested mtDNA Southern blots with a 1.8 kb COXII probe^23^.

Isolation of yeast mtDNA and analysis of mtDNA metabolic intermediates in neutral 2-dimensional agarose gels (2D NAGE) was performed as previously described^10^. Duplicate or triplicate samples were analyzed. The 1.8 kb a11 *rho*− mtDNA was digested with XbaI that cleaves once in the repeat. S1 treatment was performed with 0.2 U S1/ 1 μg DNA for 1 min at 37 °C. The blots were detected with a ^32^P-labelled probe covering the full-length a11 repeat. Analysis of mtDNA copy-number and mtDNA topology in a11 irc3Δ mutants was performed using DNA isolated from yeast cells that after gene disruption had been cultivated for 100 ± 10 doublings.

### Analysis of respiratory competence of the yeast cells

The number of respiratory competent yeast cells in a glucose containing medium was measured essentially as previously described^23^. Synthetic complete -Leu media containing 2% glucose or glycerol as a carbon source were used for yeast propagation in order to avoid the loss of plasmids.

## Additional Information

**How to cite this article**: Gaidutšik, I. *et al.* Irc3 is a mitochondrial DNA branch migration enzyme. *Sci. Rep.* **6**, 26414; doi: 10.1038/srep26414 (2016).

## Supplementary Material

Supplementary Information
