# Supplementary material for: Irc3 is a mitochondrial DNA branch migration enzyme
Source: Sci Rep. 2016 May 19;6:26414. doi: 10.1038/srep26414 (PMC4872236; doi:10.1038/srep26414)
Supplement: Supplementary Information [file srep26414-s1.pdf]

Irc3 is a mitochondrial DNA branch migration enzyme

Ilja Gaidutšik, Tiina Sedman, Sirelin Sillamaa and Juhan Sedman

Institute of Molecular and Cell Biology, University of Tartu, Riia 23b, Tartu 51010, Estonia

Supplementary Figure 1
Gaidutšik et al 2015

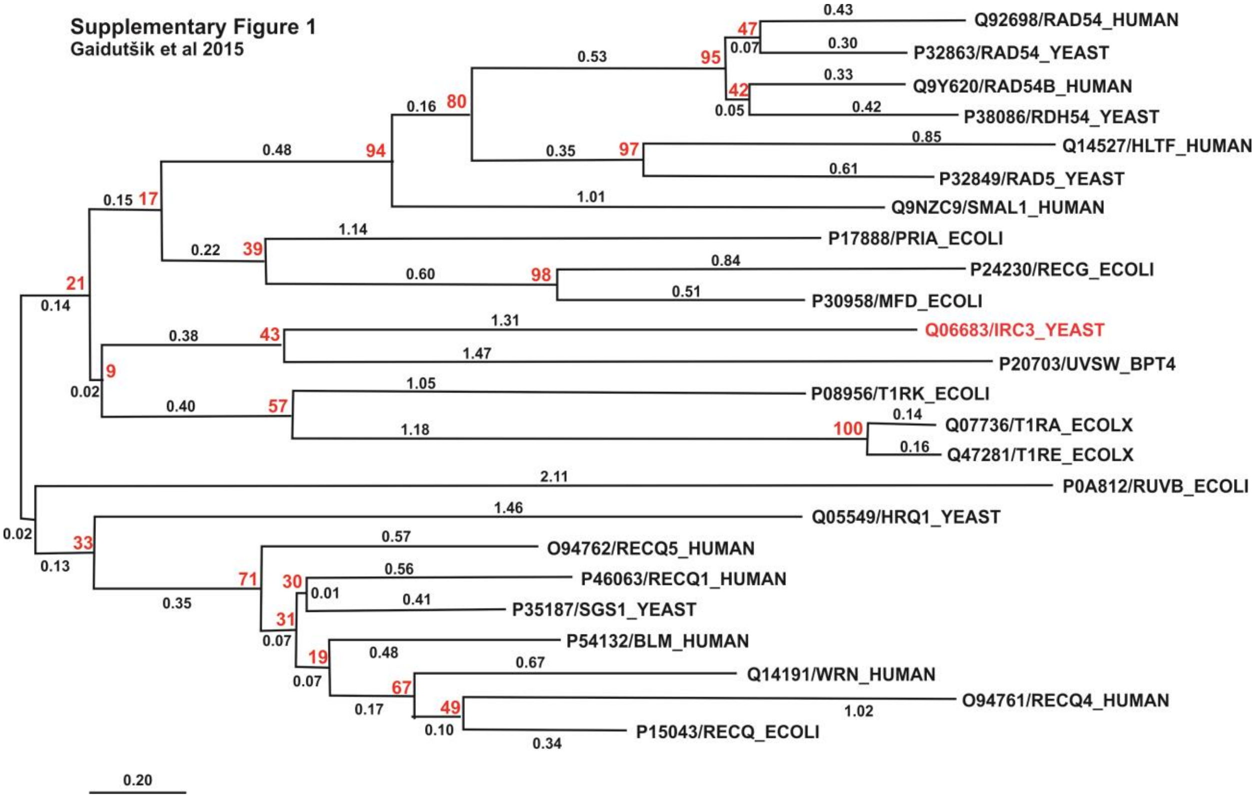

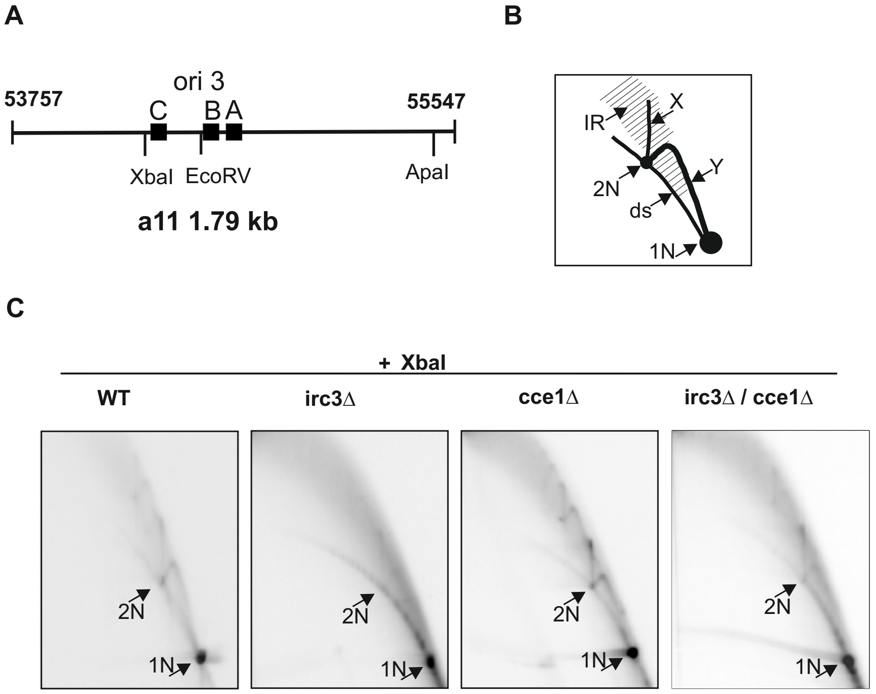

Supplementary Figure 2
Gaidutšik et al 2016

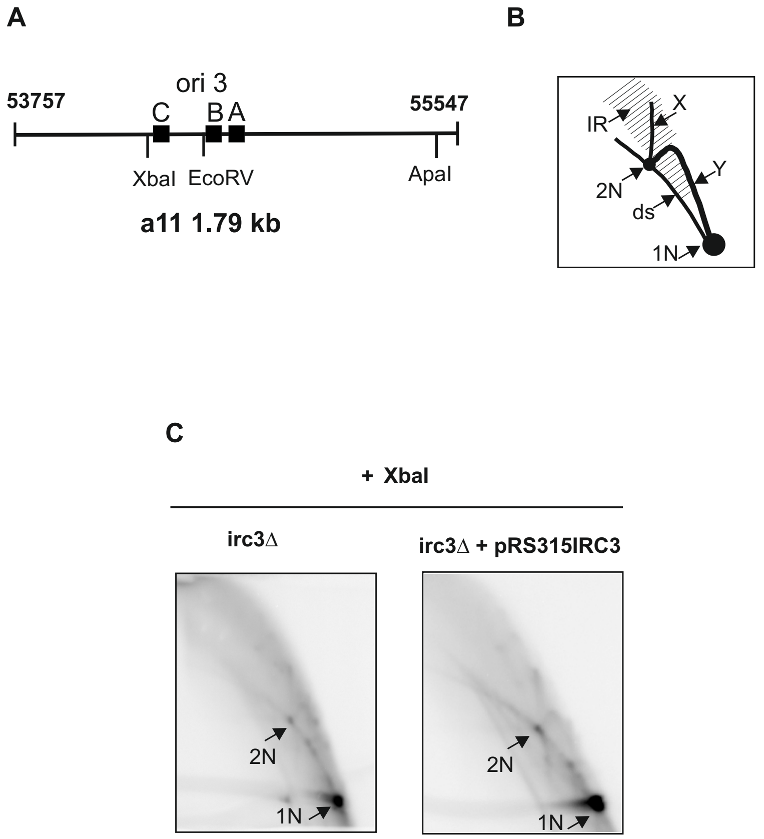

Supplementary Figure 3
Gaidutšik et al 2016

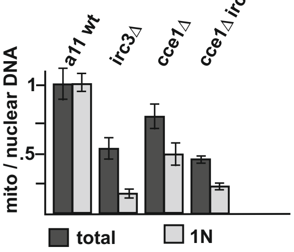

Supplementary Figure 4
Gaidutsik et al 2016

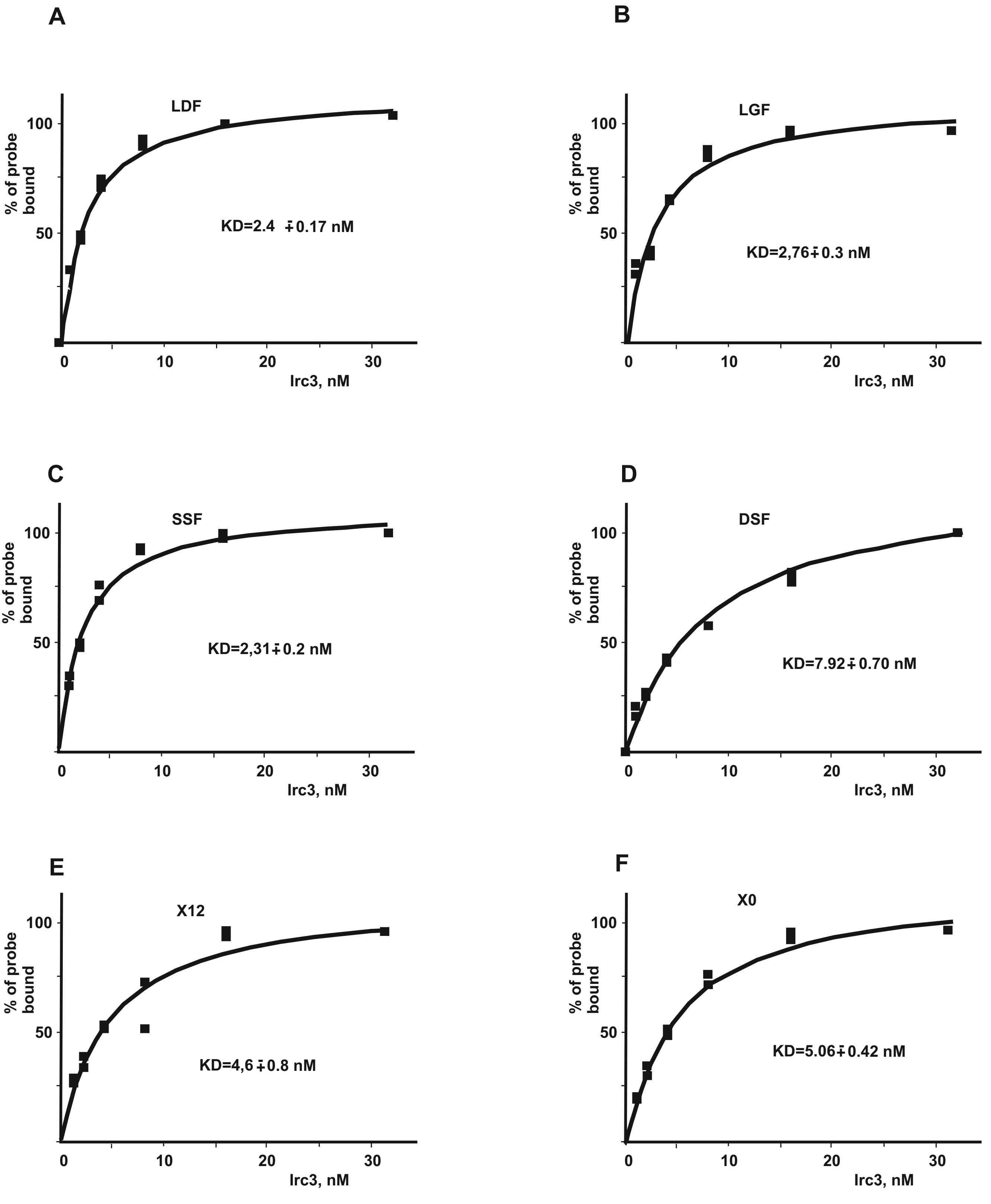

Supplementary Figure 5
Gaidutšik et al 2016

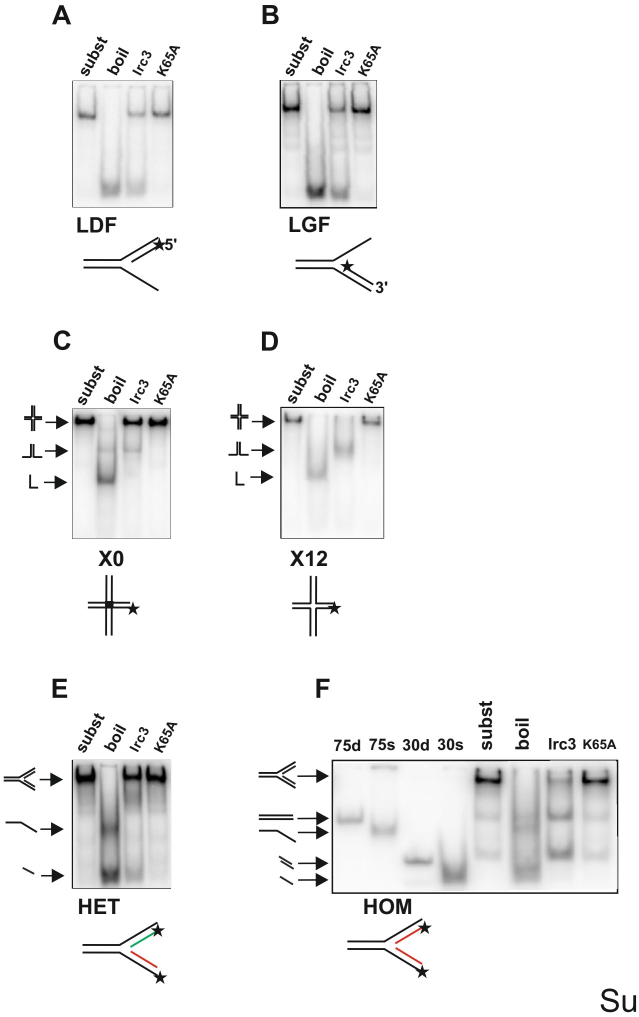

Supplementary Figure 6
Gaidutsik et al 2016

Supplementary Figure 1 Legend. Evolutionary relationships of selected SF II enzymes. Shown are the SF II enzymes with branch migration or fork regression activity in *E. coli*, *H. sapiens* and *S. cerevisiae*; Irc3 of *S. cerevisiae* and the Class I Restriction enzymes motor subunits, T1RA, T1RE and TRX as the closest homologs to Irc3 among *E. coli* cellular helicases.
The alignment for the tree is made with MUSCLE alignment software (1). The evolutionary history was inferred using the Neighbor-Joining method (2). The optimal tree with the sum of branch length = 23,84433129 is shown. The percentage of replicate trees in which the associated taxa clustered together in the bootstrap test (100 replicates) are shown next to the branches (3). The tree is drawn to scale, with branch lengths (next to the branches) in the same units as those of the evolutionary distances used to infer the phylogenetic tree. The evolutionary distances were computed using the JTT matrix-based method (4) and are in the units of the number of amino acid substitutions per site. The analysis involved 24 amino acid sequences. All positions containing gaps and missing data were eliminated. There were a total of 165 positions in the final dataset. Evolutionary analyses were conducted in MEGA6 (5).

Supplementary Figure 2 Legend. A duplicate 2D blot demonstrating the role of Irc3 in the modulation of rho- mtDNA. A different experiment of 2D analysis replicating the main Figure 1. (A) A map of the 1.8 kb a11 rho- mitochondrial DNA (mtDNA) repeat containing actively transcribed *ori3* with characteristic A, B and C boxes. The coordinates correspond to the reference yeast mtDNA sequence (Genbank: KP263414.1). (B) A scheme of 2D-gel electrophoresis analysis. ds: linear double-stranded molecules; x: X-arc; y: Y-arc; 1N: unit size (1.8 kb) molecules; 2N: double-unit size (3.6 kb) molecules, IR: irregular branched DNA molecules. (C) 2D analysis of mtDNA isolated from log phase cultures of the a11 strain with the indicated nuclear mutations. mtDNA was digested with XbaI

Supplementary Figure 3 Legend. Reintroduction of Irc3 into a11 irc3Δ cells. (A) A map of the 1.8 kb a11 rho- mitochondrial DNA (mtDNA) repeat containing actively transcribed *ori3* with characteristic A, B and C boxes. The coordinates correspond to the reference yeast mtDNA sequence (Genbank: KP263414.1). (B) A scheme of 2D-gel electrophoresis analysis. ds: linear double-stranded molecules; x: X-arc; y: Y-arc; 1N: unit size (1.8 kb) molecules; 2N: double-unit size (3.6 kb) molecules, IR: irregular branched DNA molecules. (C) 2D analysis of mtDNA isolated from log phase cultures of the a11 irc3Δ strain and a11 irc3Δ IRC3+strain. mtDNA was digested with XbaI. Both yeast strains were grown in SC-Leu medium and harboured a centromeric Leu2 plasmid pRS315 or a pRS315 derivative containing the IRC3 gene.

Supplementary Figure 4 Legend.Mitochondrial DNA copy-number analysis in the indicated a11 mutants. Relative mtDNA copy-number was analysed in the indicated yeast strains of a11 background as described in Materials and Methods.

Supplementary Figure 5 Legend. Irc3 binding to different branched DNA molecules. Irc3 binding to different DNA substrates was measured using the EMSA assay as described in the

Materials and Methods (Main Text). The tests were performed in duplicates using the following concentrations of Irc3 protein: 0.5 nM, 1 nM, 2 nM, 4 nM, 8 nM, 16 nM. The binding data were analyzed using nonlinear regression analysis and were fitted to a one binding site model using Graph Pad Prism software. The DNA substrates are described in the Main Text and in Supplementary Table 2. The measured Kd values and the corresponding standard deviation values are indicated for each DNA substrate on individual panels.

Supplementary Figure 6 Legend. Irc3 ATPase defective mutant K65A is inactive in unwinding, fork reversal and branch migration assays. A-F. Unwinding/branch migration assay with Irc3 or Irc3 mutant K65A. Reaction were performed as described in the Main Article using 5 mM wt or mutant Irc3. Lanes: subst – 2,5nM substrate incubated without protein, boil – 3´90˚C treated substrate. Irc3 – 5nM wt Irc3. K65A – 5nM K65A Irc3. Schematic representations of substrates are indicated bewlow the lanes. **A-B.** Leading and lagging fork substrates. Labelled oligo is marked with asterisk. **C-D.** X0 and X12 substrates. Labelled oligo is marked with asterisk. Schematic representations of different products and substrates are on the left. **E.** HET substrate. Schematic representations of different products and substrates are on the left. Green – heterologous oligonucleotide. **F.** Hom substrate. Schematic representations of different products, substrates and labelled oligonucleotide markers are on the left. 75d – first double stranded product of fork reversal reaction, 75s – single stranded oligonucleotide, 30d – second double stranded product of fork reversal reaction, 30s – single stranded oligonucleotide. Red – complementary oligonucleotides forming a double stranded product (30d).

Supplementary References

1. Edgar RC (2004). "MUSCLE: multiple sequence alignment with high accuracy and high throughput". Nucleic Acids Research 32 (5): 1792–1797.
2. Saitou N. and Nei M. (1987). The neighbor-joining method: A new method for reconstructing phylogenetic trees. Molecular Biology and Evolution 4:406-425.
3. Felsenstein J. (1985). Confidence limits on phylogenies: An approach using the bootstrap. Evolution 39:783-791.
4. Jones D.T., Taylor W.R., and Thornton J.M. (1992). The rapid generation of mutation data matrices from protein sequences. Computer Applications in the Biosciences 8: 275-282.
5. Tamura K., Stecher G., Peterson D., Filipski A., and Kumar S. (2013). MEGA6: Molecular Evolutionary Genetics Analysis version 6.0. Molecular Biology and Evolution30: 2725-2729.

Supplementary Table 1. List of oligonucleotides used in this study.

Oligo Name	Substrate	Sequence 5´-3’	Length (base)	Used for
49A	49DS	GACGCTGCCGAATTCTGGCTTGCTAGGACATCTTTGCCCACGTTGACCC	49	ATPase<br>EMSA
49B	49DS	GGGTCAACGTGGGCAAAGATGTCCTAGCAAGCCAGAATTCGGCAGCGTC	49	ATPase<br>EMSA
R1	3´ over; SSF; DSF; LDF; LGF	GTCGGATCCTCTAGACAGCTCCATGATCACTGGCACTGGTAGAATTCGGC	50	ATPase<br>EMSA<br>unwinding
R2	5´ over; SSF; DSF; LDF; LGF	CAACGTCATAGACGATTACATTGCTACATGGAGCTGTCTAGAGGATCCGA	50	ATPase<br>EMSA<br>unwinding
R3	5´ over; DSF; LGF	TAGCAATGTAATCGTCTATGACGTT	25	ATPase<br>EMSA<br>unwinding
R4	3´ over; DSF;LDF	TGCCGAATTCTACCAGTGCCAGTGAT	26	ATPase<br>EMSA<br>unwinding
H1	HET; HOM	GTTTTCCCAGTCACGACGATGCTCCGGTACTCCAGTGTAGGCATATTAC<br>GAATTCTTGAGGCAGGCATGGTAGCT	75	unwinding
H2	HOM	AGCTACCATGCCTGCCTCAAGAATTCGTAATATGCCTACACTGGAGTAC<br>CGGAGCATCGTCGTGACTGGGAAAAC	75	unwinding
H3	HET	GATCGTTGCATTCATTCTGGAGGCCTACGGTATGCCTACACTGGAGTAC<br>CGGAGCATCGTCGTGACTGGGAAAAC	75	unwinding
H4	HET;HOM	AGCTACCATGCCTGCCTCAAGAATTCGTAA	30	unwinding
H5	HOM	TTACGAATTCTTGAGGCAGGCATGGTAGCT	30	unwinding
H6	HET	CCGTAGGCCTCCAGAATGAATGCAACGATC	30	unwinding
X1	X0	GTCGGATCCTCTAGACAGCTCCATGATCACTGGCACTGGTAGAATTCGGC	50	ATPase<br>EMSA<br>unwinding
X2	X0	CAACGTCATAGACGATTACATTGCTACATGGAGCTGTCTAGAGGATCCGA	50	ATPase<br>EMSA<br>unwinding
X3	X0	TGCCGAATTCTACCAGTGCCAGTGATGGACATCTTTGCCCACGTTGACCC	50	ATPase<br>EMSA<br>unwinding
X4	X0; X12	TGGGTCAACGTGGGCAAAGATGTCCTAGCAATGTAATCGTCTATGACGTT	50	ATPase<br>EMSA<br>unwinding
X5	X12	GACGCTGCCGAATTCTGGCTTGCTAGGACATCTTTGCCCACGTTGACCC	49	ATPase<br>EMSA<br>unwinding
X6	X12	CAACGTCATAGACGATTACATTGCTAGGACATGCTGTCTAGAGACTATCGA	51	ATPase<br>EMSA<br>unwinding

X7	X12	ATCGATAGTCTCTAGACAGCATGTCCTAGCAAGCCAGAATTCGGCAGCGT	50	ATPase assay<br>EMSA<br>unwinding

Supplementary Table 2. Model DNA substrates

Schematic representation	Substrate name	Oligos	Labelled oligo(s)
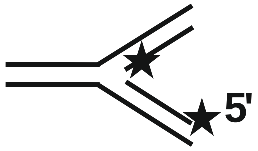	DSF	R1, R2, R3, R4	R3, R4
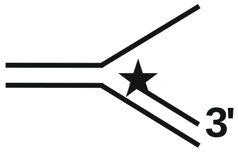	LGF	R1, R2, R3	R3
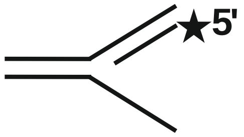	LDF	R1, R2, R4	R4
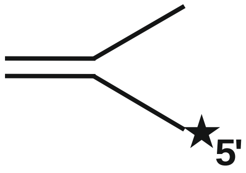	SSF	R1, R2	R2
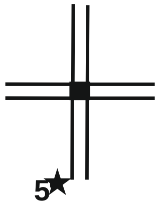	X0	X1, X2, X3, X4	X4

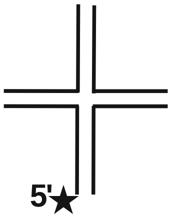	X12	X5, X6, X7, X4	X4
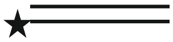	49DS	49A, 49B	49A
	49SS	49A	49A
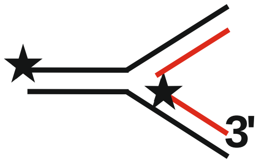	HOM	H1, H2, H4, H5	H2, H4
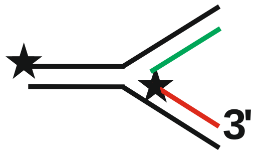	HET	H1, H6, H3, H4	H3, H4
